# Supplementary material for: Impact of Atmospheric Polycyclic Aromatic Hydrocarbons (PAHs) of Falling Dust in Urban Area Settings: Status, Chemical Composition, Sources and Potential Human Health Risks
Source: Int J Environ Res Public Health. 2023 Jan 10;20(2):1216. doi: 10.3390/ijerph20021216 (PMC9858625; doi:10.3390/ijerph20021216)
Supplement: Supplementary file 1 [file ijerph-20-01216-s001.zip › ijerph-2081524-supplementary.pdf]

Table S1. Description of measured polycyclic aromatic hydrocarbons (PAHs).

PAH Name	Symbol used in this study	Number of rings
Naphthalene	P_1	2
Acenaphthylene	P_2	3
Acenaphthene	P_3	3
Fluorene	P_4	3
Phenanthrene	P_5	3
Anthracene	P_6	3
Fluoranthene	P_7	4
Pyrene	P_8	4
Benzo(b+j)fluoranthene	P_9	5
Benzo(k)fluoranthene	P_{10}	5
Benzo(a)pyrene	P_{11}	5
3-Methylcholanthrene	P_{12}	4
Dibenz(a.h)acridine	P_{13}	5
Indeno(1.2.3-cd)pyrene	P_{14}	6
Dibenz(a.h)anthracene	P_{15}	5
Benzo(g.h.i)perylene	P_{16}	6
